# Parkinson’s Disease Dementia: Synergistic Effects of Alpha-Synuclein, Tau, Beta-Amyloid, and Iron

**DOI:** 10.3389/fnagi.2021.743754

**Published:** 2021-10-11

**Authors:** Jiajun Han, Yaohua Fan, Peipei Wu, Zifeng Huang, Xinrong Li, Lijun Zhao, Yichun Ji, Meiling Zhu

**Affiliations:** ^1^Traditional Chinese Medicine Innovation Research Center, Shenzhen Hospital of Integrated Traditional Chinese and Western Medicine, Guangzhou University of Chinese Medicine, Shenzhen, China; ^2^Shenzhen Bao’an Traditional Chinese Medicine Hospital, Guangzhou University of Chinese Medicine, Shenzhen, China

**Keywords:** beta-amyloid, tau, alpha-synuclein, iron, Parkinson’s disease dementia

## Abstract

Parkinson’s disease dementia (PDD) is a common complication of Parkinson’s disease that seriously affects patients’ health and quality of life. At present, the process and pathological mechanisms of PDD remain controversial, which hinders the development of treatments. An increasing number of clinical studies have shown that alpha-synuclein (α-syn), tau, beta-amyloid (Aβ), and iron are closely associated with PDD severity. Thus, we inferred the vicious cycle that causes oxidative stress (OS), due to the synergistic effects of α-syn, tau, Aβ, and, iron, and which plays a pivotal role in the mechanism underlying PDD. First, iron-mediated reactive oxygen species (ROS) production can lead to neuronal protein accumulation (e.g., α-syn andAβ) and cytotoxicity. In addition, regulation of post-translational modification of α-syn by iron affects the aggregation or oligomer formation of α-syn. Iron promotes tau aggregation and neurofibrillary tangles (NFTs) formation. High levels of iron, α-syn, Aβ, tau, and NFTs can cause severe OS and neuroinflammation, which lead to cell death. Then, the increasing formation of α-syn, Aβ, and NFTs further increase iron levels, which promotes the spread of α-syn and Aβ in the central and peripheral nervous systems. Finally, iron-induced neurotoxicity promotes the activation of glycogen synthase kinase 3β (GSK3β) related pathways in the synaptic terminals, which in turn play an important role in the pathological synergistic effects of α-syn, tau and Aβ. Thus, as the central factor regulating this vicious cycle, GSK3β is a potential target for the prevention and treatment of PDD; this is worthy of future study.

## Introduction

Parkinson’s disease (PD) is a common neurodegenerative disease in middle-aged and older people. The main symptoms of PD include asymmetric resting tremor, bradykinesia, postural instability, and rigidity. Patients with PD often experience cognitive deficits in the course of the disease and may develop symptoms of dementia in the late stage of PD. At the initial diagnosis of PD, more than 20% of patients show cognitive impairment, and about 80% eventually develop Parkinson’s disease dementia (PDD; Aarsland et al., 2017; Goldman and Sieg, 2020). According to the Movement Disorder Society Task Force, the recommended criteria for PDD diagnosis include fulfilling the set of diagnostic criteria for PD, PD developed prior to the onset of dementia, PD associated with a decreased global cognitive efficiency, a cognitive deficiency that is severe enough to impair daily life, and impairment in more than one cognitive domain (Dubois et al., 2007). At present, the process and pathological mechanisms of PDD remain unclear. The primary pathological feature of PDD is the accumulation of misfolded alpha-synuclein (α-syn) aggregates that form intraneuronal Lewy bodies (LBs) that are predominantly localized to presynaptic terminals. Previous studies have reported that cortical, diffuse, or limbic LBs and Lewy neurites are closely associated with dementia in PD (Gomperts, 2016; Jellinger and Korczyn, 2018; Milán-Tomás et al., 2021), and cortical LBs and neuropathy in PDD were found to be more severe than those seen in PD. In addition to LBs, Alzheimer’s disease (AD)-type pathology is also closely associated with the pathological mechanism of PDD and is severe in more than 50% of patients with PDD (Irwin et al., 2012). A recent study has suggested that AD-type pathology, which is characterized by the superposition of beta-amyloid (Aβ) and hyperphosphorylated tau, plays a synergistic role in PDD (Irwin et al., 2012). Moreover, levels of tau, neurofibrillary tangles (NFTs), and Aβ plaques have been found to be positively correlated with cognitive impairment (Irwin et al., 2013). The combination of LBs and AD-type pathology is considered to have a strong pathological association with PDD (Irwin et al., 2013). Clinical studies have shown that PDD is associated with severe α-syn pathology in the hippocampus and entorhinal and occipitotemporal cortexes, and Aβ pathology and tau pathology in the striatum (Smith et al., 2019; Kouli et al., 2020; Howard et al., 2021). The combination of LBs, Aβ, and tau pathologies has a strong correlation with the pathology in patients with PDD (Compta et al., 2011). However, the underlying synergistic effects of α-syn, tau, and Aβ in PDD are still unclear.

Iron plays an important role in promoting nervous system development and maintaining neuronal function (Hsieh et al., 2020; Donker et al., 2021). Brain iron is essential for neurotransmission, myelination of neurons, DNA synthesis, gene expression, and mitochondrial respiration. Iron deficiency in the brain during childhood can lead to psychomotor disorders and delayed language and physical development (Thirupathi and Chang, 2019). In addition, the content of iron in the brain increases with age (Ashraf et al., 2018). Iron metabolism disorders have been associated with many neurodegenerative diseases, such as AD, PD, PDD, Friedreich’s ataxia, and multiple sclerosis (Costa-Mallen et al., 2017; Xuan et al., 2017; Chen et al., 2021; La Rosa et al., 2021; Tham et al., 2021). Several clinical studies have shown that iron accumulation in the substantia nigra (SN) and several basal ganglia structures is partly associated with cognitive decline in patients with PD and PDD (Rossi et al., 2014; Costa-Mallen et al., 2017; Genoud et al., 2017; Xuan et al., 2017; Gao et al., 2020). Excessive iron can induce oxidative stress by producing reactive oxygen species (ROS), especially hydroxyl free radicals (Ward et al., 2014; Thirupathi and Chang, 2019). Previous work has found that the specific insoluble amyloid plaques and NFTs in dementia contain high concentrations of iron, which are about three times more than those found in normal people (940 μM compared with 340 μM), and that the local accumulation of iron can lead to neuronal dysfunction (Roberts et al., 2012). Iron abnormalities are related to misfolding of Aβ produced by amyloid precursor protein (APP) and hyperphosphorylated tau (found in plaques and tangles; Derry et al., 2020). Abnormal iron metabolism and α-syn misfolding are prevalent in PD (Song and Xie, 2018), and there is also abnormal accumulation of Aβ and tau in PDD (Irwin et al., 2012). Therefore, exploring the interactions between Aβ, tau, α-syn, and iron could further our understanding of the pathological mechanism underlying PDD.

## The Interaction Between Aβ, Tau, α-Syn, and Iron

### The Interaction Between α-Syn and Iron

#### Iron, Directly and Indirectly, Promotes α-Syn Aggregation

**Figure 1:**
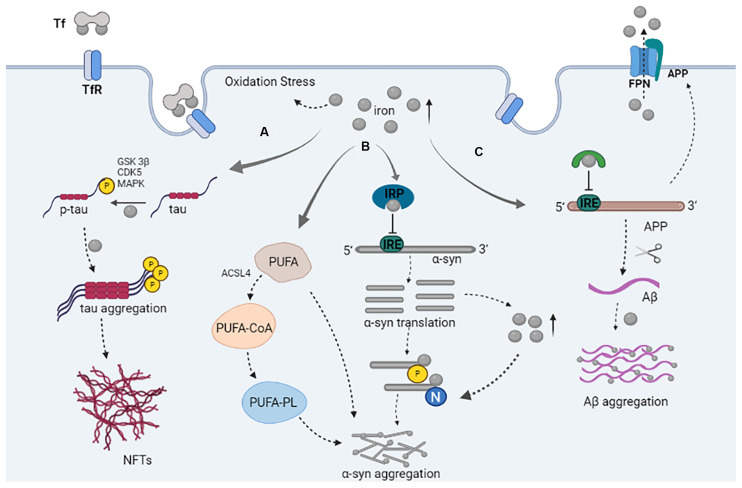
The role of iron in the pathology of tau, α-syn, and Aβ. **(A)** Iron and tau kinase (GSK3β, CDKs, MAPK) play a role in the phosphorylation of tau; iron promotes tau aggregation and NFTs formation. **(B)** The binding of IRP to iron inhibited its binding to IRE, resulting in increased expression of α-syn; α-syn can promote an increase in iron levels; iron participates in the post-translational modification of α-syn and promotes the aggregation of α-syn. Iron can promote the increase of PUFA; PUFA are conjugated to CoA by ACSL4 allowing PUFA-CoA to be incorporated into the phospholipids (PL); free or phospholipid-bound PUFAs promote the aggregation of α-syn. **(C)** The binding of IRP to iron inhibits its binding to IRE, which results in increased APP expression. The synergistic effect of APP and FPN causes iron outflow; Aβ produced from APP processing; iron promotes Aβ aggregation.

Aggregated α-syn, a landmark pathological characteristic of PDD, is encoded by the SNCA gene. The physiological role of α-syn is implicated in various cellular processes, such as maintaining the synaptic function, affecting neurotransmitter storage, and neurotransmitter release within the synapse (Nemani et al., 2010; Duce et al., 2017). SNCA gene duplication, triplication, or mutation aggravates the accumulation of misfolded α-syn, which can form insoluble fibrils through oligomerization (Hijaz and Volpicelli-Daley, 2020). In addition, α-syn aggregation has been found in cells treated with iron, which suggests that iron also plays a key role in inducing α-syn aggregation (Figure 1). Furthermore, iron-induced α-syn aggregation is dose-dependent, and the accumulation of iron in the SN during aging or LB disease may increase the aggregation rate of α-syn (Ostrerova-Golts et al., 2000).

Using a focused monolayer fluorescence technique, previous work has found that Fe^3+^ significantly increased the aggregation of α-syn and induced the formation of large oligomers at low micromolar concentrations *in vitro*, due to direct interaction with amyloid fibrils (Kostka et al., 2008; Levin et al., 2011). A previous study demonstrated that even physiological concentrations of iron can form α-syn oligomer species at nanomolar protein concentrations (Nübling et al., 2014). *In vitro* experiments could already show that iron can bind to α-syn and is able to foster α-syn aggregation (Davies et al., 2011; Perfeito et al., 2014; Jansen van Rensburg et al., 2021), a recent study has found that iron enrichment occurring in particular areas may also affect α-syn spreading pathology (Dauer Née Joppe et al., 2021). Li et al. (2020) found that the accumulation of iron ions in the brains of patients with PD accelerates the prion-like proliferation of α-syn fibrils in a vicious cycle. Iron ions first promote the cellular internalization of the fibrils, and the internalized fibrils further aggregate to the natural α-syn seeds, and, finally, the mature fibrils are released into the extracellular space to induce further propagation (Li et al., 2020). The accumulation of both α-syn and iron in the SN establish the local conditions required for α-syn-mediated hydrogen peroxide formation and its conversion to hydroxyl radicals *via* the Fenton reaction, and this leads to the death of the vulnerable nigral neurons (Turnbull et al., 2001; Vasquez et al., 2017).

A recent study has found that iron can also promote the aggregation and transmission of α-syn by inhibiting autophagosome-lysosomal fusion (Xiao et al., 2018). The 5′ untranslated region of α-syn transcripts contains an iron response element (IRE; Zhou and Tan, 2017). Iron regulatory proteins (IRP) can bind to the IRE under iron deficiency conditions, thereby inhibiting protein synthesis, whereby increased iron levels cause IRP to bind to iron, which promotes the dissociation of IRP from the α-syn mRNA, leading to facilitation of the translation of the α-syn mRNA (Anderson et al., 2012; Zhou and Tan, 2017). Qu et al. (2019) demonstrated that rosmarinic acid might protect against iron-induced cellular neurotoxicity *via* the ability of the IRP/IRE system to inhibit the expression of α-syn and induce the degradation of α-syn. Increased iron levels lead to increased translation of the α-syn protein, and the over-expression of α-syn can promote the formation of aggregates by increasing the proportion of partially folded α-syn (Uversky, 2007). This further indicates that iron is a key contributor to α-syn aggregation.

In addition to directly promoting the aggregation of α-syn, iron can also indirectly promote the aggregation of α-syn through lipid metabolism. Given that iron is a cofactor to the family of lipoxygenase enzymes, an elevated labile iron pool catalyzes the formation of phospholipid hydroperoxides in the plasma membrane, which determines cellular sensitivity to ferroptosis (Mahoney-Sánchez et al., 2021). Free cytosolic polyunsaturated fatty acids (PUFA) are conjugated to coenzyme-A (CoA) by acyl-CoA synthetase long-chain family member 4 (ACSL4), which allows PUFA-CoA to be incorporated into the phospholipids in the plasma membrane (Doll et al., 2017). When exposed to free or phospholipid-bound PUFAs, α-syn will increase the propensity to aggregate and oligomerize (De Franceschi et al., 2009; Sánchez Campos et al., 2018).

### Iron Regulates the Post-translational Modification of α-Syn

There are many post-translational modifications of α-syn, including phosphorylation, acetylation, ubiquitination, oxidation, nitration, and truncation (Figure 1). Multiple post-translational modifications can change the structure of α-syn and regulate its physiological function. This may be related to aggregation or oligomer formation of α-syn (Barrett and Timothy Greenamyre, 2015). The following contents will focus on the relationship between nitration and phosphorylation of α-syn and iron, and iron-induced oxidative stress.

#### Modification of the Phosphorylation of α-Syn by Iron

Phosphorylation is the most common and important protein modification associated with the pathology of α-syn. Several phosphorylation sites have been identified on α-syn, either at serine, tyrosine, or threonine, such as S129, S87, Y39, and Y125. Approximately 90% of α-syn deposited in LBs is phosphorylated at S129, while under normal physiological conditions, only 4% or less of α-syn was phosphorylated (Fujiwara et al., 2002; Anderson et al., 2006); this suggests that phosphorylation can affect α-syn aggregation. Phosphorylation typically occurs at S129. The involved kinases include polo-like kinase (PLKs), G protein-coupled receptor kinase (GRKs), casein kinase (CKs), and leucine-rich repeat kinase 2 (LRRK2; Qing et al., 2009; Oueslati, 2016; Rafael Guimarães et al., 2021). Iron can induce the up-regulation of α-syn and pS129 α-syn (S129 phosphorylation) through polo-like kinase 2 (PLK2) and casein kinase 2 (CK2; Wang et al., 2019). Fe^3+^ may promote pS129 α-syn oligomer formation, because Fe^3+^ not only prevents pS129 α-syn from combining with the membrane but also facilitates protein interactions *via* the additional negative charges introduced to the protein structure through phosphorylation (Nübling et al., 2014). It is also possible that iron plays a role in promoting the phosphorylation of S129. Electron microscope results have revealed that iron can change the morphology of α-syn fibrils and accelerate the aggregation of wild-type and mutant α-syn (Li et al., 2020). In addition to S129, there are other phosphorylation sites on the tyrosine residue of α-syn, such as Y125 and Y39 (Brahmachari et al., 2016; Zheng et al., 2019). The modified Y125 can increase the phosphorylation level of S129 through casein kinase 1 (CK1; Kosten et al., 2014), and a reduction of the Y125 modification can also reduce the phosphorylation level of S129, thereby reducing the abnormal aggregation of α-syn caused by phosphorylation of S129 (Fayyad et al., 2020). Antioxidants can completely block the iron-induced kinase up-regulation and α-syn phosphorylation, which indicates that there is a close association between these processes and oxidative stress (Wang et al., 2019).

#### Nitration of α-Syn by Iron

α-Syn nitration modification is widespread in AD, PD, LB dementia, multiple system atrophy, and other neurodegenerative diseases (Chavarría and Souza, 2013). One study found that there were more nitrated α-syn-positive dopaminergic cell bodies in aged monkeys than in adult monkeys, and 20% of the α-syn positive dopaminergic neurons were co-immunoreactive with nitrated α-syn, which suggests that nitrated α-syn accumulated in SN neuronal bodies of aging primates (McCormack et al., 2012). Nitration often occurs at the four tyrosine residues of α-syn (Tyr-39, Tyr-125, Tyr-133, and Tyr-136; Burai et al., 2015). Nitration enhancement of α-syn Tyr-39 contributes to methamphetamine-induced dopaminergic toxicity (Qiao et al., 2019). Nitration of Tyr-125 contributes to the formation of α-syn dimers (Takahashi et al., 2002). Nitration of Tyr-39 and the formation of di-tyrosine involving this residue play a key role in the oligomerization of α-syn. The Tyr-39 mutation will lead to multiple di-tyrosine cross-linking and interfere with the formation of higher-order aggregates. Tyr-39 and Tyr-125 mutations also increase dimer and monomer levels (Burai et al., 2015), which leads to an increase in α-syn neurotoxicity.

Nitrated α-syn can induce adaptive immune responses and may intensify microglia activation (Reynolds et al., 2009). Activated microglia can induce oxidative stress in dopaminergic neurons, which leads to neuronal nitration and dopaminergic neuron death (Shavali et al., 2006; Yu et al., 2010). Recent studies have found that myeloperoxidase (MPO) is expressed in neurons of patients with PD and a humanized MPO-A53T mouse model of PD. MPO is co-located with neurons nitrated with α-syn, carbamylated with α-syn, and hypochlorous acid-modified with α-syn, and is associated with α-syn aggregation and motor damage. Furthermore, MPO inhibitors have beneficial effects in the treatment of neurodegenerative diseases such as PDD (Maki et al., 2019). Bibenzyl compound 20C, a Chinese medicinal extract, can inhibit the adaptive immune response mediated by nitration of α-syn (Wang et al., 2021), which may inhibit oxidative stress and neuronal death. Iron-induced oxidative stress is the most common harmful reaction, and excessive iron content is associated with an increase in nitration stress, which leads to increases in the tyrosine nitration level. There is extensive nitrative damage in neurodegeneration with brain iron accumulation type 1, intracellular nitrative insult will cause the accumulation of α-syn (Paxinou et al., 2001). In animals and patients with iron overload, excessive iron can promote the formation of nitrogen species (such as the hydroxyl radical, superoxide anion, and nitric oxide) that disrupt the redox balance of cells and lead to chronic nitrosative stress (Zhang et al., 2012). Therefore, iron may play an important regulatory role in α-syn nitration.

### α-Syn Affects Iron Transport and Deposition

Many studies have investigated the role of iron in α-syn aggregation and the regulation of post-translational modification of α-syn. However, α-syn has also been found to play a role in iron homeostasis. α-Syn is considered to be an iron-binding protein that can bind to Fe, Fe^3+^, or Fe^2+^ (Peng et al., 2010; Davies et al., 2011). Fe^3+^ can also be reduced to Fe^2+^ by its iron reductase activity to increase the content of Fe^2+^ in cells (Brown, 2013; Ortega et al., 2016), and oxidative stress and neuronal degeneration can be induced by the Fenton reaction. Post-translational modification of α-syn can regulate iron transport, while N-terminal acetylation of α-syn can promote dynamic protein-mediated transferrin receptor (TfR) endosome transport and iron internalization. However, phosphorylated α-syn can reduce iron input through TfR endocytosis (Duce et al., 2017). In neurons exposed to excessive iron, the overexpression of α-syn leads to an increase in intracellular iron levels and the redistribution of iron from the cytoplasm to the perinuclear region in α-syn-rich inclusions (Ortega et al., 2016). This is further evidence of the binding between iron and α-syn.

Early study has found that when cells are exposed to dopamine alone, α-syn does not cause aggregation, but iron-treated cells induce aggregation. The overexpression of α-syn makes cells sensitive to iron-mediated toxicity. It is possible that the aggregation of α-syn increases the cellular iron content, resulting in increased oxidative toxicity (Ostrerova-Golts et al., 2000). Some data have also indicated that increased intracellular concentrations of α-syn, due to, for example, gene dose effects, autophagy injury, or proteasome dysfunction, may contribute to intracellular iron accumulation in neurons exposed to excessive iron, which in turn promotes further oligomerization and α-syn aggregation. In turn, α-syn oligomerization leads to an increase in intracellular iron levels (Ortega et al., 2016), which suggests that α-syn oligomerization and iron accumulation may be a mutual promotion process. Bi et al. (2020) found that α-syn could induce p38 mitogen-activated protein kinase (MAPK) activation to phosphorylate parkin at Ser131, inactivate parkin’s E3 ubiquitin ligase activity, further inhibit divalent metal transporter 1 (DMT1) ubiquitination, and increase the expression of DMT1 protein in α-syn-overexpressed SH-SY5Y cells and mutant human A53T α-syn transgenic mice. This process aggravates oxidative stress, interferes with iron homeostasis, and participates in the deposition of iron in the SN (Bi et al., 2020). Guo et al. (2021) have reported iron deposition in the SN–striatum system of monkeys after intranasal injection of exogenous α-synuclein preformed fibrils (α-syn PFFs). One potential mechanism underlying this effect is that α-syn-PFFs treatment triggers the accumulation of iron in microglia to initiate a neuroinflammatory reaction, which in turn induces a cascade reaction between iron deposition and microglia activation, producing hydrogen peroxide and hydroxyl radicals, and releasing pro-inflammatory factors; this, in turn, leads to neuroinflammatory α-syn accumulation and dopaminergic neuron degeneration (Guo et al., 2021).

Shekoohi et al. (2021) found that reduced expression of α-syn in retinal pigment epithelial (RPE) cells significantly reduced TfR1 and ferritin levels and reduced iron deposition compared to control RPE cells. However, overexpression of α-syn significantly increased the levels of these two proteins (Shekoohi et al., 2021). α-Syn can also damage ferritinophagy, which results in the accumulation of iron-rich ferritin in RPE cells (Baksi and Singh, 2017). In addition, other studies have shown that inhibition of α-syn expression by siRNA can prevent the effects of iron on mitochondria and cell death (Ganguly et al., 2020). Extracellular α-syn can mediate endoplasmic reticulum stress to regulate cellular iron-related proteins and further affect iron metabolism (Mi et al., 2021). Therefore, a high labile iron environment and α-syn aggregation could generate toxic dopamine reactive quinones and reactive species that induce the overproduction of ROS and mitochondrial dysfunction *via* mitochondrial respiratory complex I inhibition (Duce et al., 2017; Nonnekes et al., 2018; Zhou et al., 2019). Mitochondrial dysfunction results in increased ROS production, which may also contribute to lipid peroxidation in the plasma membrane.

## The Interaction Between Tau and Iron

NFTs formed by abnormal phosphorylation of tau protein are widely found in PDD and their levels are positively correlated with the severity of dementia. Tau dysfunction and misfolding may lead to iron deposition, which relies on iron-induced oxidative damage to produce toxicity, leading to neuron loss or ferroptosis (Dixon et al., 2012). Conversely, iron can induce tau phosphorylation and tau aggregation, promote the progression of dementia, increase Aβ aggregation and APP protein expression, promote the formation of NFTs, and finally lead to neuronal death (Xie et al., 2012; Spotorno et al., 2020; Figure 1). Fe^3+^ is associated with NFTs in dementia and progressive supranuclear palsy, and induces the accumulation of hyperphosphorylated tau. Reduction of Fe^3+^ to Fe^2+^ can reverse the aggregation of tau and dissolved tau species from the brain of patients with dementia (Yamamoto et al., 2002). It has been found that excessive intake of iron can induce hippocampal iron disorder in APP and presenilin 1 (PS1) double-transgenic mice and adult rats, and promote the overexpression of Aβ and phosphorylated tau (Chen et al., 2019). Metallic lithium can lead to iron accumulation by regulating tau expression. After lithium treatment, tau levels in mouse brains decreased, while iron in the SN and cortex increased. In neuron culture, lithium reduced the outflow of iron by reducing tau proteins. Tau protein and amyloid precursor gene-knockout mice were protected from lithium-induced iron elevation and neurotoxicity (Wood, 2016; Lei et al., 2017).

Tau kinase is also involved in the interaction between iron and tau. Bautista et al. (2016) found that tau hyperphosphorylation is one of the key steps in the formation of NFTs, and can be regulated by iron through the abnormal activation of tau kinases, such as GSK3β, cyclin-dependent kinase 5 (CDK5), and MAPK (Rao et al., 2020). Guo et al. (2013) demonstrated that high iron levels can induce tau phosphorylation at Thr205, Thr231, and Ser396 sites in the brains of APP/PS1 transgenic mice, while intranasal administration of deferoxamine (DFO) in APP/PS1 transgenic mice also reduced the activity of iron-induced CDK5 and GSK3β, which in turn inhibited tau phosphorylation. Therefore, iron and DFO antagonize and regulate tau phosphorylation in a CDK5- and GSK3β-dependent manner (Guo et al., 2013).

p-tau expression has been found to decrease after injection of an GSK3β inhibitor in MPTP mice (Hu et al., 2020). However, treatment with ebselen, a DMT1 inhibitor, has been found to inhibit CDK5 and GSK3β activity in ferrous-treated SH-SY5Y cells, which eventually reduces hyperphosphorylation of tau (Xie et al., 2012). APP can also interact with iron transporters on the neuron surface to regulate iron output. Tau loss leads to the accumulation of immature APP in the endoplasmic reticulum and prevents APP from being transported to the neuron surface, which leads to the toxic accumulation of iron (Lei et al., 2012; Bassil, 2013). Hephaestin (a transmembrane iron oxidase) has been recently identified as an active agent in the complex of APP and iron transporter proteins (Dlouhy et al., 2019). Ceruloplasmin also has the property of iron oxidase and can interact with iron transporters (Honarmand Ebrahimi et al., 2013).

Iron chelators reduce tau phosphorylation, and iron overload impairs the brain mitochondrial balance and increases brain oxidative stress, which leads to the loss of apoptotic dendrites and ultimate increases in AD-like lesions. Iron chelators, such as deferiprone (DFP) and DFO, can protect the brain following iron overload by reducing the accumulation of brain iron, the destruction of brain mitochondrial kinetics, and the loss of dendrites (Sripetchwandee et al., 2016). Age-dependent brain atrophy, iron accumulation, and SN neuron loss in tau-knockout mice with cognitive deficits and Parkinson’s syndrome can be prevented by oral administration of iron chelators (Xie et al., 2012). In addition, Wan et al. (2019) found that insulin signals were involved in iron-induced abnormal phosphorylation of tau. In primary cultured neurons, Fe^2+^ causes tau phosphorylation and reduces tyrosine phosphorylation of insulin receptor β, insulin signaling substrate 1, and phosphoinositide 3- kinase p85α. Insulin treatment also reduces tau hyperphosphorylation in neurons cultured with excessive iron, which is indicative of the existence of an insulin-dependent pathway (Wan et al., 2019). Homeostasis between tau and insulin signals can be restored by activating the insulin receptor, or by targeting the downstream pathway with protein tyrosine phosphatase 1B inhibitors, GLP-1 receptor agonists, or intranasal insulin administration (Gratuze et al., 2018). Stable insulin signals may facilitate the clearance of pathological forms of tau, which could be considered in potential treatments for PDD.

## The Interaction Between Aβ and Iron

Accumulated Aβ and iron clearly play a central role in the pathology of PDD. First, extensive research has demonstrated that Aβ can bind to iron (Plascencia-Villa et al., 2016). The high affinity of Aβ for binding to iron and its ability to reduce iron levels lead to the formation of hydrogen peroxide, which causes oxidative damage (Foster et al., 2020). The ROS generated by iron-induced Aβ accumulation is also toxic to neurons (Liu et al., 2011). Animal models have shown that elevated brain iron levels lead to oxidative stress, promote Aβ toxicity and tau protein dysfunction, enhance neuronal cell death, and lead to neural degradation and cognitive dysfunction (Lane et al., 2018). In a study with patients with mild cognitive impairment, Ayton et al. (2017) found that individuals with high iron levels and a high amyloid load showed greater cognitive decline than individuals with only one of the pathological conditions. van Bergen et al. (2016) demonstrated that the progression of cognitive dysfunction was caused by cerebral iron deposition and spatial co-localization of Aβ plaques. Evidence has also suggested that iron accumulation in neurons containing NFTs and neurites adjacent to senile plaques is associated with cognitive decline (Ding et al., 2009; van Bergen et al., 2018). Low cerebrospinal fluid Aβ42 is also a predictor of future cognitive impairment in patients with PD (Lim et al., 2019). Redox-active metal ions such as iron can accelerate Aβ aggregation and induce ROS production (Ayton et al., 2017; Cheignon et al., 2018). In particular, iron can catalyze ROS production under acidic conditions *via* Fenton chemistry (Mai et al., 2017). And diffusely distributed Aβ spots were observed throughout the whole hippocampus of Young-High Fe mice (Chen et al., 2021). In addition, the interaction of iron with Aβ may result in the disorder of the iron balance (Qin et al., 2021).

Iron has not only been shown to bind to Aβ, but also to regulate APP production. Iron levels affect APP translation through iron-responsive elements in APP transcript 5′-UTR (Cho et al., 2010; Figure 1). Iron overload conditions can increase the protein expression of APP in the brain (Sripetchwandee et al., 2016). APP also plays a role in maintaining neuronal iron homeostasis. Cell surface APP stabilizes the iron export protein, ferroportin (FPN), and allows cellular iron to flow out through the porin (McCarthy et al., 2014; Venkataramani et al., 2018). Choi et al. (2021) demonstrated that treadmill exercise promoted α-secretase-dependent APP processing through low iron-induced enhancement of furin activity to reduce cognitive decline and Aβ-induced neuronal cell death, accompanied by a decrease in the levels of lipid peroxidation products and an increase in the ability of antioxidant defense enzymes. However, APP modified after translation, such as phosphorylation and glycosylation, can change its transport to the cell surface, and then affect its binding to FPN, which leads to imbalanced iron homeostasis (Tsatsanis et al., 2019). In addition, the intracellular processing of APP may impair iron output by destabilizing cell surface iron transporters. By contrast, non-amyloid processing of APP on the cell surface promotes the stability of iron transporters and thus reduces iron levels in neurons (Tsatsanis et al., 2020). Therefore, changes in intracellular transport that are related to changes in APP processing may lead to neuronal iron level increases and oxidative stress in dementia pathology.

A recent study has suggested that a high-iron diet boosted Aβ expression compared to mice fed normal diets. The iron concentration and ferritin in the hippocampus of APP/PS1 mice fed with a high-iron diet were significantly increased, the activity of superoxide dismutase was significantly decreased, and Aβ1–42 protein expression was higher. In addition, iron deposition and Aβ plaques were gathered in the hippocampal DG region, accompanied by cognitive dysfunction (Chen et al., 2019). It could be seen that an iron overload diet induced the disorder of iron in the mouse hippocampus and Aβ overexpression. Lipocalin 2 is involved in many physiological processes such as inflammation, iron metabolism, and cell death and may contribute to the pathophysiology of neurodegenerative diseases such as AD and PD. Ferroptosis is also involved in the pathogenesis of PD (Tian et al., 2020; Li et al., 2021; Si et al., 2021). Iron chelating agents such as DFO and DFO can inhibit the production of proteins such as Aβ-induced lipocalin 2 in primary cultured astrocytes, which may contribute to the protection of nerves (Dekens et al., 2020). Overexpression of hepcidin in astrocytes significantly improved Aβ 25–35 induced cellular damage in the cerebral cortex and hippocampus. It is possible that iron FPN1 acts on brain microvascular endothelial cells, which in turn reduce Aβ25–35-induced oxidative stress and apoptosis, and ultimately protects cells from damage (Zhang et al., 2020).

### Parkinson’s Disease Dementia: the Synergistic Effects of α-syn, Tau, Aβ, and Iron

The increase in iron content in the brain may be related to the increase of hyperphosphorylated tau, Aβ, and α-syn (Lu et al., 2017). Additionally, p-tau, Aβ, and α-syn work together to promote the pathogenesis of PDD (Figure 1 and Table 1). In one study, APP transgenic mice exhibited severe hyperphosphorylated tau-positive neurites and synaptic dystrophy near amyloid plaques (Radde et al., 2006). Li et al. (2015) injected Aβ oligomers into the hippocampus of young and old tau gene knockout mice and found that tau ablation prevented Aβ-induced cognitive impairment, hippocampal neuron loss, and iron accumulation. Therefore, tau protein may be a downstream mediator of Aβ toxicity (Li et al., 2015). In addition, phosphorylation of tau can occur at many sites, and the hyperphosphorylation of tau protein at Thr181 has been associated with an increased expression of Aβ and APP proteins, as well as the progression of neurodegeneration (Spotorno et al., 2020). Tau-hyperphosphorylation of the Thr181 amino acid may be a specific biomarker of brain pathological states, such as AD (Saman et al., 2012). α-Syn and Aβ have also been found to have a certain synergistic effect. Bassil et al. (2020) injected α-syn PFFs into mice with abundant Aβ plaques. Aβ deposition significantly accelerated the pathogenesis of α-syn and spread throughout the whole brain. Notably, hyperphosphorylation of tau, partial neuronal loss, motor, and cognitive impairment were also noted in α-syn PFFs-injected 5xFAD mice. This may indicate the presence of a “pre-feedback” mechanism, whereby Aβ plaques enhance the dissemination and diffusion of endogenous α-syn following injection of PFFs (Bassil et al., 2020). Swirski et al. (2014) found a correlation between levels of S129-phosphorylated α-syn and Aβ. When SH-SY5Y cells transfected with the α-syn gene were exposed to aggregated Aβ42, the proportion of phosphorylated α-syn at S129 was significantly increased, which indicated an association between α-syn and Aβ (Swirski et al., 2014).

**Table 1:** Changes of α-syn, tau, Aβ, and iron in PDD.

**Subject**	**α-syn**	**tau**	**Aβ**	**Iron**	**Summary finding**	**References**
**A53T transgenic monkey**	**↑**				High α-syn correlated with increased rate of cognitive decline	Niu et al. (2015)
**PDD flies**	**↑**				α-Syn increases in PD model with cognitive dysfunction	Fatima and Siddique (2019)
**Combined Lewy–Alzheimer transgenic mice**	**↑**	**↑**	**↑**		Aβ, tau, and α-syn can synergistically exacerbate the aggregation and deposition of each other, thereby promoting the additional acceleration of cognitive decline	Clinton et al. (2010)
**5xFAD mice injected with α-syn**	**↑**		**↑**		α-Syn PFFs injections into the brains of 5 × FAD mice accelerated cognitive decline in the Y-maze as early as 3 mpi, and cognitive performance progressively worsened with time	Bassil et al. (2020)
Prnp-SNCA*A53T mice (overexpressing mutated α-syn A53T mice)	**↑**	**↑**			Tau oligomers and α-syn are increased in the brains of Prnp-SNCA*A53T mice, indicating a cognitive function deficit, based on the inability to discriminate between novel and familiar objects	Gerson et al. (2018)
**PDD C57BL/6 mice**			**↑**		Increased Aβ is associated with decreased cognitive function	Ba et al. (2019)
**PDD rat**	**↑**	**↑**			Accumulation of α-syn and tau hyperphosphorylation are associated with decreased cognitive function. The injured insulin signaling pathway may be involved in this dopamine-dependent cognitive impairment	Yang et al. (2020)
**PDD C57BL/6 mice**	**↑**		**↑**		α-Syn and Aβ pathology are associated with the cognitive status of PDD	Choi et al. (2019)
**overexpressing human α-syn rat**	**↑**				The rats overexpressing human α-syn exhibited spatial learning and memory deficits in the Morris water maze task	Hall et al. (2013)
**Mice expressing human wild-type α-syn under the Thy1 promoter**	**↑**				Overexpressing human α-syn mice exhibit deficits in cholinergic systems involved in cognition, and cognitive deficits in domains affected in early PD	Magen et al. (2012)
**PD patients**				**↑**	Brain tissue iron, measured using quantitative susceptibility mapping, can track cognitive involvement in PD. This may be useful to detect signs of early cognitive change	Thomas et al. (2020)
**PDD patients and PD patients**				**↑**	Higher iron deposition is also observed in the unilateral hippocampus of the PDD patients when compared to non-demented PD patients. Moderate correlation of iron content in the PD and PDD patients with both cognitive and other neuropsychiatric impairment is found.	Li et al. (2018)

**Figure 2:**
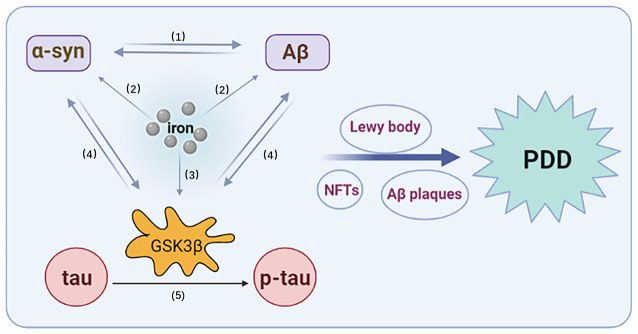
Interaction of tau, α-syn, Aβ, iron, and GSK3β. (1) The synergistic effect of α-syn and Aβ. (2) Iron promotes the overexpression of α-syn and Aβ. (3) Iron promotes the activation of GSK3β. (4) α-Syn and Aβ can promote an increase in GSK3β, which can, in turn, promote the expression of α-syn and Aβ. (5) GSK3β promotes the phosphorylation of tau.

In the synaptic terminals of adult and aged rats exposed to free iron, GSK3β phosphorylation has been reported to be enhanced and GSK3β-related signaling pathways activated (Uranga et al., 2009), while DFO treatment can attenuate the activation of GSK-3β (Zhang et al., 2015). Activated GSK3β signals may also play a role in the pathological synergy of tau, Aβ, and α-syn. First, the phosphorylation of tau by GSK-3β enhances metal ion-induced oligomer formation and co-aggregation with α-syn (Nübling et al., 2014). Overexpression of human P301L mutant tau promotes the phosphorylation and dimerization of endogenous α-syn by activating GSK-3β in rTg4510 mice (Takaichi et al., 2020). Tau not only promotes the synthesis and phosphorylation of α-syn, but also increases the insoluble form of α-syn and enhances its toxicity. Furthermore, α-syn promotes the hyperphosphorylation of GSK3β-dependent tau by reducing the phosphorylation of Ser9 and increasing the phosphorylation of Tyr216. Inhibition of GSK3β reduces the phosphorylation and accumulation of α-syn in PD pathology (Gąssowska et al., 2014). Yang et al. (2018) found that Aβ exposure induced GSK3β activity, while GSK3 proteins (including GSK3α and GSK3β) promoted Aβ production and stimulated apoptotic signals *via* Aβ. Both α-syn and Aβ overexpression were associated with increased levels of active GSK3β, which led an increased hyperphosphorylation of tau. Finally, inflammation associated with GSK3β activation was found to lead to apoptosis (Yang et al., 2018). Therefore, the synergistic effects among iron, tau, α-syn, Aβ, and GSK-3β are involved in the pathophysiology of PDD (Figure 2).

Colom-Cadena et al. (2013) found that the density of MAPT·H1 haplotypes and amyloid deposits was related to the number of Aβ and α-syn deposits in the brains of patients with dementia with LBs. Aβ deposition has also been pathologically associated with synuclein PD and PDD cases. In addition, misfold-mediated heterotypic binding induced by the binding of α-syn and tau K18 has been reported to synergistically promote the formation of Aβ (Bhasne et al., 2018). Lu et al. (2020) found that two variants of monomers α-syn and tau (tau23 and K19) synergistically promoted amyloid fibril formation, causing them to co-aggregate *in vitro*. Nuclear magnetic resonance spectroscopy experiments showed that α-syn directly interacted with tau23 and K19 by virtue of its highly negatively charged carbon terminal. The C-terminal deletion effectively eliminated its association with tau23 and K19, and its synergistic effect of promoting fibrillation (Lu et al., 2020).

## Discussion

PDD is a common occurrence in PD and has a significant impact on the health and daily life of patients. Although most evidence has indicated an α-syn pathology in the development of PDD, there is a notable heterogeneity in symptomatology and timing of dementia between PDD cases. An increasing number of studies have shown that LBs, AD-type pathology, and iron dysmetabolism appear to contribute to the emergence of PDD (Smith et al., 2019; Kouli et al., 2020; Howard et al., 2021). It is possible that α-syn is transmitted from degenerating neurons into neighboring neurons and non-neuronal cells; indeed, α-syn aggregates have been found in the SN, the nucleus basalis of Meynert, cerebral cortex, locus coeruleus, cranial nerve motor nuclei, and locus coeruleus (Braak et al., 2006; Brundin and Melki, 2017). Astrocytes, which are the most abundant glial cells in the central nervous system, are responsible for the extracellular homeostasis of ions and neurotransmitters that are able to internalize extracellular α-syn (Lee et al., 2010, 2014). Moreover, Aβ has been found to induce an inflammatory response and iron accumulation in astrocytes, both *in vivo* and *in vitro* (Urrutia et al., 2013). Iron chelators inhibit the Aβ-induced production of lipocalin 2 in cultured astrocytes. Thus, astrocytes are a key participant in PDD pathogenesis and treatment (Zhu et al., 2019; Cui et al., 2020).

While some studies have suggested that the interaction of iron, α-syn, tau, and Aβ is a key pathological mechanism underlying PDD, the specific mechanism is not yet clear (Compta et al., 2011). According to a literature search, we found that iron can interact with α-syn, tau, and Aβ, and promote their aggregation. Iron indirectly affects disease-related proteins through oxidative stress or regulation of post-translational modifications. Conversely, α-syn, tau, and Aβ also affect iron metabolism and iron accumulation (Figure 1). Furthermore, α-syn and Aβ can promote an increase of GSK3β, which can, in turn, promote the expression of α-syn and Aβ, as well as the phosphorylation of tau (Figure 2). Thus, GSK3β plays a significant regulatory role in the synergistic effects of α-syn, tau, Aβ, and, iron, and further verification of its function in astrocytes is required. In addition, GSK3β may represent a potential biomarker of incipient cognitive decline in patients with PDD. Drug development for the prevention of GSK3β expression could also be considered in future research. For instance, immune-based therapies that target GSK3β could be an effective disease-modifying therapy in PDD. However, timing the initiation of this therapy is a critical and uncertain issue.

Recent studies on PD therapies have examined the role of the gut-brain axis, given the spread of LBs in the central nervous system and the peripheral nervous system, and particularly the enteric nervous system (Scheperjans et al., 2015; Surmeier et al., 2017; Diwakarla et al., 2021; Borah et al., 2021). Many studies have found that a high-iron diet can lead to iron disorders and overexpression of Aβ and phosphorylated tau, and iron chelating agents such as DFP and DFO can play a preventive and therapeutic role, as well as a neuroprotective role (Guo et al., 2013; Sripetchwandee et al., 2016; Rao et al., 2020). Moreover, Zederone could reduce the accumulation of amyloid plaques and improve cognitive capacity *via* the brain-gut axis. The above results suggest that targeted regulation of iron metabolism disorders *via* the brain-gut axis is a candidate treatment strategy to prevent PDD.

## Author Contributions

MZ and YF designed the article. JH, PW, ZH, XL, and LZ collected the article’s materials. JH and PW wrote the manuscript. MZ, YF, and YJ revised and polished the article. All authors contributed to the article and approved the submitted version.

## Conflict of Interest

The authors declare that the research was conducted in the absence of any commercial or financial relationships that could be construed as a potential conflict of interest.

## Publisher’s Note

All claims expressed in this article are solely those of the authors and do not necessarily represent those of their affiliated organizations, or those of the publisher, the editors and the reviewers. Any product that may be evaluated in this article, or claim that may be made by its manufacturer, is not guaranteed or endorsed by the publisher.

## Abbreviations

ACSL4: Acyl-CoA synthetase long-chain family member 4
AD: Alzheimer’s disease
APP: Amyloid precursor protein
Aβ: Beta-amyloid
CDK5: Cyclin-dependent kinase 5
CK: Casein kinase
CK1: Casein kinase 1
CK2: Casein kinase 2
CoA: Coenzyme-A
DFO: Deferoxamine
DFP: Deferiprone
DMT1: Divalent metal transporter 1
FPN: Ferroportin
GRKs: G protein-coupled receptor kinase
GSK3β: Glycogen synthase kinase 3β
IRE: Iron response element
IRP: Iron regulatory proteins
LBD: Lewy body disease
LBs: Lewy bodies
LRRK2: Leucine-rich repeat kinase 2
MAPK: Mitogen-activated protein kinase
MPO: Myeloperoxidase
NFTs: Neurofibrillary tangles
PD: Parkinson’s disease
PDD: Parkinson’s disease dementia
PLK2: Polo-like kinase 2
PLKs: Polo-like kinase
PS1: Presenilin 1
PUFA: Polyunsaturated fatty acids
ROS: Reactive oxygen species
RPE: Retinal pigment epithelial
SN: Substantia nigra
TfR: Transferrin receptor
α-syn: Alpha-synuclein
α-syn PFFs: α-synuclein preformed fibrils.
